# Down deep in the holler: chasing seeds and stories in southern Appalachia

**DOI:** 10.1186/1746-4269-9-69

**Published:** 2013-09-27

**Authors:** James R Veteto

**Affiliations:** 1 Department of Anthropology, University of North Texas, 1155 Union Circle Drive # 310409, Denton, TX 76203-5017, USA

**Keywords:** Agrobiodiversity, Appalachia, Cherokee, Ethnoecology, Ethnography

## Abstract

This essay, which is the third in the series “Recollections, Reflections, and Revelations: Ethnobiologists and their First Time in the Field”, is a personal reflection by the researcher on his experience and involvement in kinship and friendship networks while conducting agrobiodiversity research in southern Appalachia, USA. Vignettes are given from moving moments spent with Native spiritual leaders, backcountry mountain people, and local co-collaborators in the research process. The author demonstrates how lasting field friendships have helped lead to groundbreaking ethnoecological research.

## Setting the stage for Appalachian fieldwork through experience and approach

Everything informal in southern Appalachia runs through kinship and friendship networks. There is a certain humility and down-to-earthness necessary if you want to get anything done in the region. Anything that is perceived as being too “high falutin” (local slang for pretentious or pompous) will likely be met with silence, sideways contempt, or information that will result in false leads. By and large, Appalachia is a region with a hardscrabble past of poverty and struggle. Adding in a general distrust of government and state-sanctioned activities can make fieldwork challenging to the uninitiated. People who are not from Appalachia are considered “off-the-mountain” and “furriners” (foreigners) and often treated suspiciously. The first thing one must do when conducting research in southern Appalachia is to leave your ego at home and be prepared to engage in a good bit of friendly small-talk before attempting to introduce research questions. Good manners and a Southern accent help but might not come naturally to researchers from other areas of the world. Those who have never lived or spent much time in Appalachia will likely need a prolonged period of participant observation (much like any anthropological field site) to understand local norms and customs.

I had several things working for me when I first went to the field to conduct research on the agricultural biodiversity (agrobiodiversity) of Appalachia. First, I am a Southern-American with some deep historical roots in the region, although I did not grow up in Appalachia and my family migrated out of the region generations ago. Probably more importantly, I had lived in western North Carolina (in the heart of southern Appalachia) for five years after graduating with an undergraduate degree in anthropology from the University of Georgia and worked as a farmer and ethnobotanist during that time. This experience gave me some good preparation for understanding “the mountain way of living” and also a precursory knowledge of local heirloom vegetable and fruit varieties. I also had a deep bioregional commitment to learning the “old ways” of Appalachia in order to apply them to my own lifestyle in environmentally appropriate ways. These experiences turned out to be helpful in my fieldwork experience but could have been harmful, had I not let go of some of my environmentalist essentialisms of ‘pure and simple’ traditional Appalachian living. Appalachians, like most people worldwide, are complex and multi-faceted in approaches to their environment and it takes a lot of time to unravel their worldviews through listening to local stories and narratives.

## Field vignettes: stories of friendship and heirloom seeds^a^

### Encountering kinship and friendship networks in Yancey County, North Carolina

As is often the case with snowball sampling methods, I was referred to some of my best informants in a rather happenstance way when I was searching for heirloom gardeners to interview during my initial field research in western North Carolina (I did master’s thesis research in 2005 and dissertation research in 2008, along with other sporadic fieldwork trips from 2003–2008 and ongoing). For example, I was chatting with a friend and former co-worker at the Arthur Morgan School in Celo, North Carolina when I mentioned off-hand the type of research I was undertaking. He said he knew exactly who I should visit: the Williams family who live up on Bald Mountain. He gave me a phone number and told me to tell them that David sent me. In conversation with other local people about this particular family, it came out that they were known as being pretty ‘wild’ and several mentioned that a brother in the family had accidently been shot and killed on the family property some years back. This put a little bit of uneasiness in my mind, but mostly I did not worry about it as some of my closest acquaintances assured me that the Williams family were actually really good people.

As is usual in my Appalachian fieldwork experience, it took many phone calls and several weeks of attempts before I finally got a meeting set up with several members of the Williams family. Driving up to the Williams property required going far off the nearest state highway, over a high mountain pass, and following a creek-side road up a steep hill to their holler (Appalachian for “hollow” and a local word for narrow creek and river valleys that end in a mountain side, so are only accessible from one direction). I was met by a chorus of barking hunting dogs and a tall mustached Appalachia man with Levis jeans, a denim shirt, and baseball cap upon my arrival. “Howdy, you must be Jim”, he said. “Yessir, I am,” I replied. He invited me around side of the house, through a sliding-glass door, and into their living room where two women were sitting on couches.

For the first half-hour we sat around talking and getting to know one another. Central to our discussion was how my friend David (the former co-worker who had given me the Williams’ contact information) was doing and what he had been up to recently. They were curious how I knew David and what my relationship to him was. Being introduced through a mutual friend, as I have since learned in later years, is an asset in developing field rapport. Not long after our interview started and we were discussing the virtues and uses of Roughbark Candyroaster Squash (*Cucurbita maxima* Duch.), Brown Bunch Beans (*Phaeseolus vulgaris* L.), and Coxx Special Field Corn (*Zea Mays* L.), Eddie’s (the first man I had met upon arriving at the Williams household) brother Harry arrived from his house further up the holler and we were introduced. Once Harry was told by Eddie that I was friends with David, Harry heartily said, “Any friend of David is a friend of ours”. This familiarity went a long ways toward making the interview one of the best I have ever conducted. We sat for two-and-a-half hours talking about every heirloom variety of vegetable and fruit they had ever grown and looked at seed samples (the interview was conducted during the winter so we could not tour their garden during that visit). It turns out that between the Williams brothers they were maintaining twenty-eight distinct varieties of heirloom, open-pollinated vegetables and fruits, one of the highest totals that I have recorded among any family of Appalachian gardeners. To put this discovery in context, it is the same number of heirloom varieties that I recorded being grown among six *communities* in the Sierra Madre Occidental Mountains of Northwest Mexico.

As we continued to talk throughout the afternoon I found out that they were kin (second cousins) to Darnell McCrary, one of the most well-known herbalists and traditional basket-makers in southern Appalachia. Darnell was also known as being quite a lively and wily character and not prone to interacting with outsiders (at least in his advanced age) unless there was some friendly or kinship connection. Harry gave me Darnell’s phone number and told me to tell him that Harry and Eddy Williams had sent me. They assured me that Darnell was a real ‘old-timer’ and that he and his wife kept a big garden every year. About a month passed and I finally got Darnell to agree to an interview on the basis of the recommendation from the Williams brothers. When I arrived at Darnell’s residence, an older man with snow white hair and a high-pitched mountain accent answered the door and demanded to know who I was. Somewhat sheepishly, I replied that I was the guy from the university who was sent by the Williams brothers and was interested in old-timey vegetable seeds. After some more small-talk and uneasiness on my part, he let me into his living room where his wife and several male family members were sitting around. This close-knit family atmosphere is fairly normal for a visit to an Appalachian homestead in my experience—except for the fact that the two males were cleaning shotguns. This put me a little on edge but everything seemed cordial and they proceeded to ask how Eddy and Harry were doing. I told them about my visit to the Williams homestead and they proceeded to tell me all kinds of wild stories, which I will not repeat here because they sometimes ventured into realms beyond political correctness or even anthropological morals.

Darnell kept putting me off whenever I made mention of conducting an interview. After a round of stories he suggested that we go out to his field and harvest a mess (Appalachian for ‘a lot’) of ‘Creasy Greens’ (*Barbarea verna* Mill.). I was given a pocket knife and shown how to harvest the plant just at the crown of the root in order to get all the leaves together. Darnell also showed me a lot of the other medicinal plants growing around his homestead and told me traditional Appalachian uses for each. He took me to his canning shed, which contained three entire walls of canned beans, corn, beets, tomatoes, beans, apples, sauerkraut, and many other foodstuffs. Darnell told me, “If the world collapses, we won’t starve”. To this day, I have never seen a canning shed or root cellar as full as his was. When we got back inside he also told me how to cook Creasy Greens in the traditional Appalachian manner with fatback and sent me home with a fine mess to cook with my family. We still harvest and prepare Creasy Greens the way Darnell taught me.

We sat around for another hour or so in Darnell’s backcountry Tennessee house (he had moved just over the state line from Bald Mountain, North Carolina some years back), discussing all the different heirloom seeds (n = 12) that had been passed down in his family and he maintained over the years. He gave me a sample of Big Greasy Bean (*P. vulgaris* L.), a delicious Appalachian pole bean that has a slick ‘greasy’ appearance due to the lack of fine hairs on the pods, that I still grow and enjoy every garden season. Darnell never would let me turn on a recorder, as his trust in me as an outsider did not extend that far despite a reference from second cousins who were friends of a friend. Nonetheless, it was an excellent and insightful interview that taught me many intricacies about ‘old-time’ (and contemporary) Appalachian culture that I had not known previously. When I got back to my car, I scribbled everything he had told me down in my field notebook while the information was still fresh in my mind.

**Figure 1:**
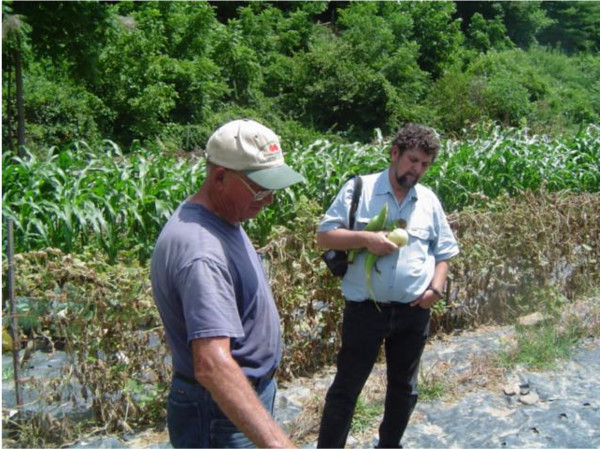
Ethnobiologist Gary Nabhan (right) with an Appalachian gardener.

My relationship with the Williams family has helped lead me down several fruitful avenues in my career as a researcher. The fact that they, along with their extended family as evidenced by Darnell, were maintaining such high agrobiodiversity levels in the most industrialized nation of the world began to show me just how important the southern/central Appalachian foodshed is as a site of agrobiodiversity conservation. When my friend and collaborator (and significant mentor), ethnobiologist Gary Paul Nabhan, came to visit during my dissertation fieldwork, we went out to see Harry. He met Gary and I in front of his house with his wife, filled up a cooler with Budweiser beer, and had us hop on the back (standing up on the rear fender and holding onto the roll bars) of his camouflaged four-wheeler as he drove up to his garden, out to several old apple trees on the property (including the ‘Mud Hole’ apple, *Malus pumila* Mill. which had never been documented anywhere else), and to see a rare grove of American Chestnut trees (*Castanea dentate* (Marshall).Borkh.). Taking cues from the Williams family and other Appalachian gardeners and orchardists that we visited on the trip (Figure 1), Nabhan decided to focus on Appalachia as the next target region for intensive study by the Renewing America’s Food Traditions alliance (RAFT). Subsequent results from my dissertation [1] and the RAFT publication *Place-Based Foods of Appalachia: From Rarity to Community Restoration and Market Recovery*[2] has shown that southern/central “Apple-achia” is the most diverse foodshed currently known in the US, Canada, and northern Mexico. It is not too far-fetched to say that my friendship with the former maintenance coordinator at the school where I was formerly Director of the farm and garden program (he used to keep our old garden tiller running with his immense mechanical skills)—and his friendship with the Williams family—played an important role in events leading to the production of a groundbreaking ethnoecological study.

### Friendship and kinship among the Eastern Band of Cherokee Indians

**Figure 2:**
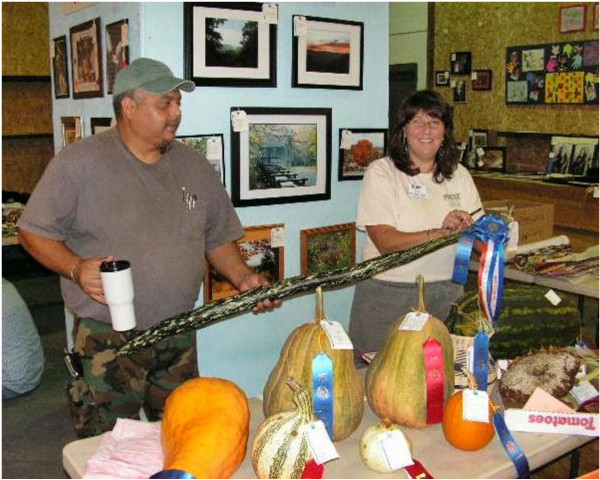
**Kevin Welch (left) and Sarah McClellan-Welch attaching awards ribbons at the Cherokee Indian Fair Agricultural Exhibit.** They are holding a snake gourd (*Trichosanthes cucumerina* L.).

**Figure 3:**
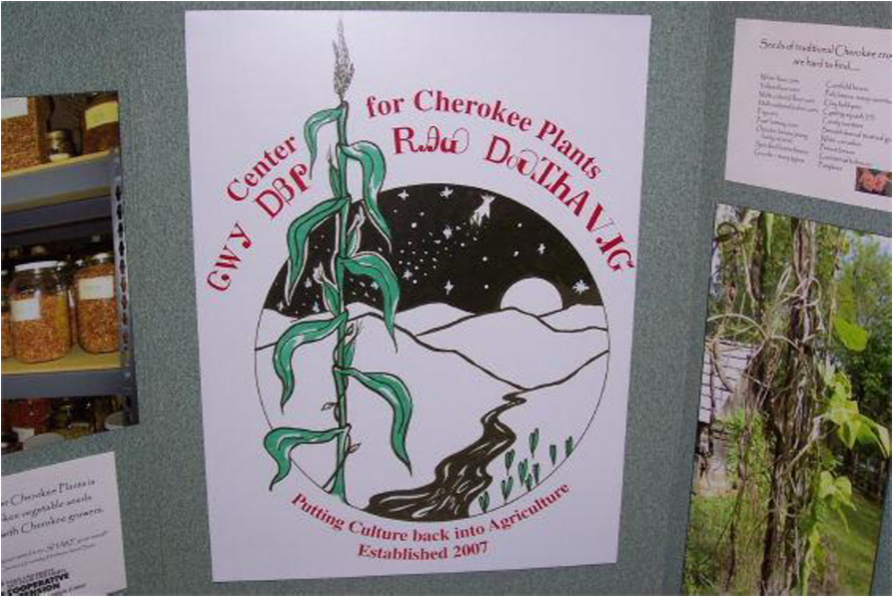
Official logo of the Center for Cherokee Plants.

As a group of original Appalachian agriculturalists, I felt it was essential to study agrobiodiversity among the Eastern Band of Cherokee Indians (EBCI) to gain greater understanding of southern Appalachia heirloom foodways during my dissertation field research. Doing research among the Cherokee, however, has different requirements than doing research among non-Native American Appalachian communities. The EBCI are a federally recognized American Indian tribe and permission is required by the Tribal Government before research can be undertaken. The tribe also has their own IRB (Institutional Review Board) process. My first meeting with the Cherokee cultural resources officer; Kevin Welch (real name, Figure 2), the founder of the Center for Cherokee Plants (Figure 3), and various other members of the community was set up by David Cozzo, a fellow University of Georgia alum and ethnobiologist working with a tribal organization called Renewing Traditional Cherokee Artisan Resources (RTCAR). Although my budding professional relationship and friendship with David (we had numerous mutual friends and colleagues) helped me get my foot in the door, I still had to prove myself to important members of the Eastern Cherokee tribe to get research permission. Out of respect and a long-held belief that indigenous peoples should have control over their own biological resources, I brought samples of twenty-five different heirloom varieties that I had collected throughout Appalachia with Cherokee names attached to them to share at our first meeting. Several of the varieties had disappeared on the reservation throughout the years, so the Cherokee were happy to receive them. Kevin Welch was pleased to add them to the seedbank he had recently assembled at the Center for Cherokee Plants. This start to the meeting helped get us off on a good foot, but I still had to demonstrate that my research would benefit (and not exploit) Cherokee communities and go through the proper channels to get permission for research. In previous years, much like among other American Indian tribes, research had been conducted on the Eastern Cherokee that they felt was detrimental to their people. I left the meeting uncertain whether or not I would be allowed proceed with my study but I did sense a budding friendship with Kevin that—if research permission was granted—might lead to fruitful collaboration.

**Figure 4:**
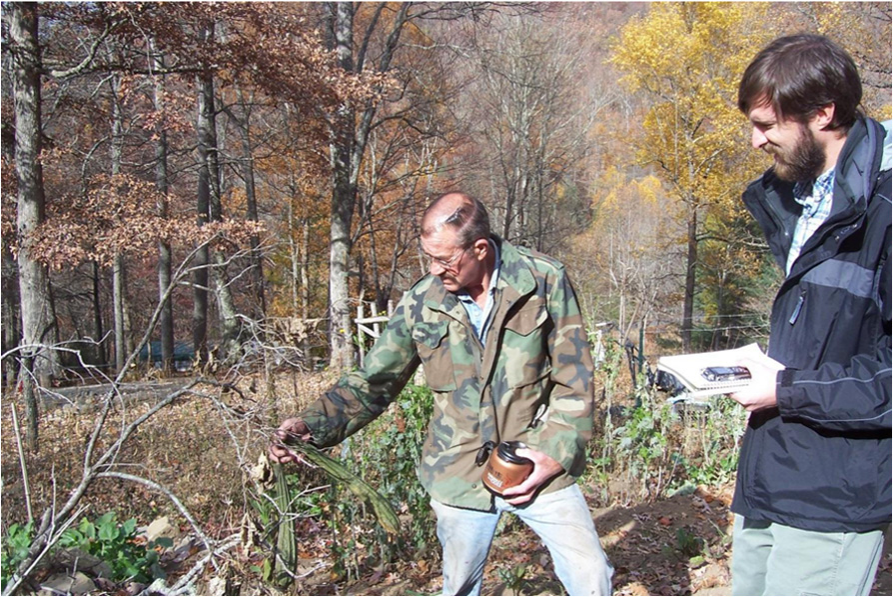
The author (right) interviewing a Cherokee gardener.

Kevin and I went ahead and started making plans for a research schedule—and just days before we were supposed to begin—permission was officially granted by the tribe. Over the next several months we visited nearly every Cherokee community among the Eastern Band to interview gardeners and farmers who were growing and maintaining traditional cultivars (Figure 4). We conducted in-depth oral history interviews with fifteen Cherokee growers and were able to document 128 distinct folk taxa among 32 plant species being grown on the Qualla Boundary (a legal title whereby tribal land is held in trust and supervised by the Bureau of Indian Affairs of the US Government, roughly equivalent to the Reservation status of the lands of most US Indian tribes). Kevin was in charge of setting up interviews, for in his own words, he knew nearly everyone in the tribe and his family network was widespread. Kevin’s lifetime of kinship and friendship networks served us well in getting interviews, many of which were with elderly gardeners who lived in deep hollers and remote communities off the back-roads of the reservation. I rode around with Kevin in his pickup truck as he told me stories of growing up as a member of the tribe, oriented me geographically with local place names and legends, and took me to special community events like the thanksgiving celebration in the traditionalist Big Cove Community, where Cherokee foods such as Bean Bread and So-chan (*Rudbeckia laciniata* L.) greens made from Cherokee crop varieties and wild foods were served.

It would have been impossible to access the Cherokee elders I interviewed had I not developed a good relationship with Kevin, which turned into a friendship that continues to this day. Kevin had already been collecting seeds and visiting Cherokee elders since he conducted a feasibility study for the Center for Cherokee Plants project in 2005. What I was able to contribute to the project was a more systematic approach developed from ethnoecological methods. Drawing on Virginia Nazarea’s cultural memory banking [3], we recorded and transcribed oral history interviews to archive with the Center for Cherokee Plants, in addition to collecting seed samples for seed banking and data from benchmark socioeconomic surveys. We also asked detailed questions about why growers were maintaining heirloom varieties in order to improve Eastern Cherokee agrobiodiversity conservation efforts and inform conservation theory and practice in general [1,4].

Kevin’s passion for valuing and preserving Cherokee traditional environmental knowledge (TEK), combined with my training and experience as an ethnoecologist and heirloom gardener, provided a mutual interest that facilitated our friendship. Finding local people who share passion for your particular ethnoecological domain and developing collaborations with them is an excellent way toward creating field relationships that can yield solid results and good feelings all around.

I will close this section by sharing two experiences that illustrate the kind of fieldwork relationships that Kevin and I were involved in. The first involved a spiritual leader of the Eastern Cherokee with whom we had been trying to set up an interview during our entire fieldwork process. Kevin was a little worried that it would not work out, as the man was very elderly and had withdrawn from interacting with the public as much as he had done in previous years. In addition, Kevin worried that relationships between his family and this elder had not always been good due to political differences and that the elder might remember an incident when Kevin was a boy that had upset him.

When we arrived at the remote residence of this elder on a crisp, cool Appalachian fall morning in 2008, he was visibly nervous that we had arrived before his daughter and other family members. We were supposed to conduct the interview in their presence as previously agreed upon. While we waited for the others to arrive, the man talked to us in a low voice while looking out the window and eventually served us some biscuits, gravy, and bacon while we waited. After about twenty-minutes, his family members arrived and we sat around talking for an hour. The first order of business was to orient Kevin and his family in relation to this elder and his family. Small talk ensued and once the elder had placed Kevin in relationship to larger kinship and community networks, we began to discuss what our study was about. Kevin’s worries were allayed as the man appeared to hold no ill will toward Kevin or his family. We were both relieved when he expressed joy and a willingness to help us on our agrobiodiversity project. As an Eastern Cherokee traditionalist, he saw a great need for preserving and promoting the gardening knowledge of the tribe, not just for conservation reasons, but also to provide a living link to the Cherokee past for youth of today growing up in a modern world. The interview with this respected elder was fascinating on many accounts and lasted for nearly three hours. In addition to gaining new insights into traditional Cherokee gardening methods and heirloom cultivars; I learned a great deal about Cherokee history, ethnography, cultural revivalism, spiritual practices, and medicinal plants. The interview had been a difficult one to arrange and required some persistence on Kevin’s part, but was certainly worth the wait.

**Figure 5:**
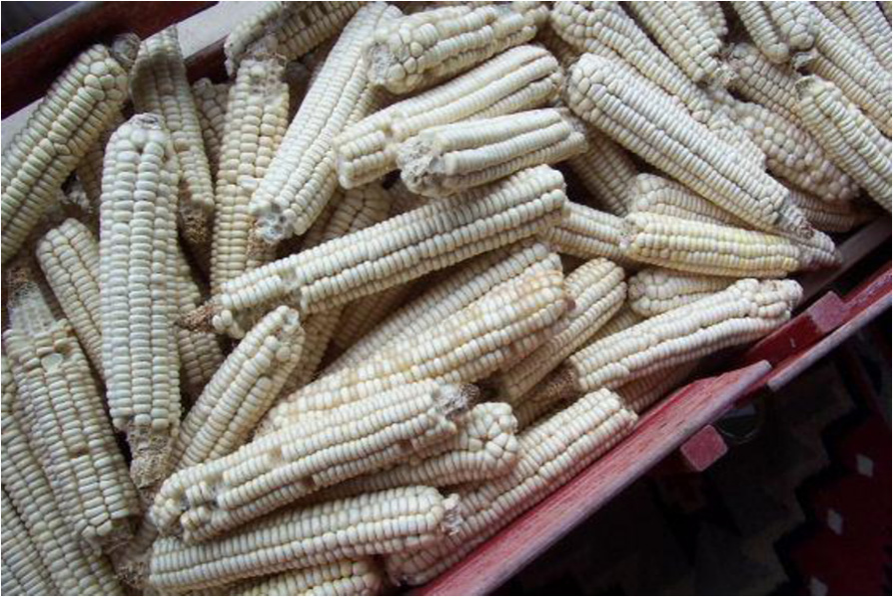
**Cherokee White Flour Corn (*Zea mays *L.).**

Another fieldwork experience demonstrated to Kevin and I the value of the work we were doing in an unexpected and tragic way. During the course of our fieldwork we interviewed a vibrant and friendly sixty-five year old Cherokee gardener who was a family friend of Kevin’s. He told us many stories of growing up on a farm, keeping an apple orchard, and growing a special corn variety to feed his flock of ducks. Of particular interest were the ears of Cherokee Yellow-flour Corn (*Zea mays* L.) that he pulled out of his storage shed and showed us. Cherokee Yellow-flour Corn is a very rare traditional variety of Cherokee corn (rarer than the more well-known Cherokee White-flour Corn—Figure 5), but several rings of kernels at the base of a few ears of the corn were of even more interest to us. Each of these kernels was a translucent milky-yellow color. Kevin immediately recognized them as being similar to the Cherokee Yellow-pearl Hominy Corn that was grown among the tribe in former years but was thought to have gone extinct. He was very excited to make this discovery and get a sample to grow seeds out at the Center for Cherokee Plants and eventually back-breed to recover Cherokee Yellow-pearl Hominy Corn. I sat back in amazement and scribbled down notes in my field notebook as the two men sat and talked about the virtues and uses of this nearly-extinct corn variety.

A few months after my fieldwork among the Eastern Cherokee was completed, Kevin got in touch with me and shared some terrible news. The energetic and healthy sixty-five year-old gardener who had given us the sample of Yellow Pearl Hominy Corn had unexpectedly died after complications resulting from eating fish. This deeply saddened me as he was one of the most pleasant people I have met in all of my fieldwork experiences. At the request of his family, Kevin asked if I could make an extra copy of the oral history interview we had recorded with the man. The family knew we had conducted an interview in the months before he passed away and did not have any other recordings of his voice. I rushed a CD copy into the mail immediately and sent it off. The incident was a powerful lesson for showing how important and meaningful this type of ethnoecological research can be. Particularly when interviewing the elderly, we are documenting important knowledge that can be lost to local people forever when individuals die. It is a truism that life, death, and change are part of a dynamic and never-ending process and that cultural loss is inevitable; but for those family members of the deceased gardener who had never thought to record his voice—and for Kevin who retrieved valuable gardening and agrobiodiversity knowledge that can benefit the Eastern Cherokee people as a whole—such work is not anachronistic and static but part of their ongoing personal and collective history. I was touched to have played a small role in making a contribution to both the family of the Cherokee gardener and the Center for Cherokee Plants.

## Reflections

For any ethnoecologist, making friends in the field is both an essential and enjoyable part of our research experience. It can be as important as doing a good literature review, constructing appropriate research questions; or carrying a notebook, tape recorder, or plant press into the field. After all, having a good research design and field instruments will not do much good if we do not have local people to interact or collaborate with. And, as is true among any human group, friendships are essential to developing relationships and social networks. However, good friendships cannot be manufactured and must develop organically, and this requires us to be genuine with other people and not unnecessarily rush the research process before appropriate relationships are developed.

In my experience conducting research, living, and working in southern Appalachia over the past fifteen years; it has been the case that some of my first meetings have created some of my most lasting relationships. I have continued to visit (sometimes with other ethnoecologists) and draw upon the experience of the Williams family periodically through the years after our first meeting in 2005. Kevin Welch has not only remained a friend, but he has made a trip to visit me as a guest speaker at The University of Georgia, our families have enjoyed the Cherokee Indian Fair together in the fall, and he and I have collaborated on several publications [4,5]. Whenever I visit Eastern Cherokee land I make a point to see Kevin. None of these relationships were planned. If I had included how my southern Appalachian fieldwork friendships would evolve in my research design, I would be a man of great foresight. I had no idea how ‘productive’ these relationships would end up being. But that was not really the point. Undertaking ethnoecological research necessitates interacting with other people—and often people of different cultural backgrounds than your own. As long as you remain open and friendly during the process, friendships are bound to develop. Which is a wonderful phenomenon in and of itself. Everything else is icing on the cake.

### Endnote

^a^Pseudonyms are used to protect the privacy of individuals involved in my fieldwork.

## Competing interests

The author declares that he has no competing interests.
